# Medical Students' Perceptions of Emergency Medicine Careers

**DOI:** 10.7759/cureus.1608

**Published:** 2017-08-24

**Authors:** Kiersten Pianosi, Samuel A Stewart, Katrina Hurley

**Affiliations:** 1 Department of Otolaryngology, University of Western Ontario; 2 Community Health and Epidemiology, Dalhousie University; 3 Emergency Department, IWK Health Centre

**Keywords:** career choice, emergency medicine, medical education, qualitative research

## Abstract

Introduction

Previous studies on specialty choice have investigated specialty characteristics that are appealing to undergraduate students. Little is known about how students’ attitudes towards Emergency Medicine (EM) careers evolve over their schooling.

Methods

An open-ended survey of medical students’ career interests was distributed five times over the four-year undergraduate curriculum from 1999 to 2008 at Memorial University. We tested specialty choices across genders, and looked at how likely a student’s choice in their first year influenced their final year choice, a metric we termed “endurance”. The qualitative data was coded to identify key themes and sentinel quotes. Lastly, we conducted semi-structured interviews with academic emergency physicians at Dalhousie University to assess the relevance of these findings to postgraduate training.

Results

Males expressed more interest in EM than females. EM had more endurance than internal medicine, but less than family medicine, over the four-year curriculum. The biggest drawbacks for EM included lack of patient follow-up and lack of EM experience; positive perspectives focused on clinical variety and elective experiences. Lifestyle was prominent, seen as both positive and negative. Emergency physicians considered EM lifestyle attractive, and characterized medical students’ perceptions as “skewed,” highlighting lack of insight into system flaws.

Conclusions

Medical students’ opinions towards EM tended to shift over time, particularly the perception of the work. Medical students’ perceptions differ from that of experienced emergency physicians. Medical schools may be able to improve clinical exposure and provide more informed counselling or mentoring with respect to EM.

## Introduction

Emergency medicine (EM) is a growing specialty in Canada. In 2003, the Canadian Resident Matching Service (CaRMS) listed 22 places in Royal College of Physicians and Surgeons of Canada (RCPSC) Emergency Medicine residency training programs. By 2015, this number grew to 68 places. In proportion to this, interest in EM as a first career choice also increased, with 28 graduating students ranking EM first in 2003, and 129 ranking it first in 2016 [1]. While undergraduates’ enthusiasm for EM is growing, evidence suggests that emergency physicians experience moderate to high levels of burnout, making it important to understand whether students have realistic expectations about an EM career [2].

Broadly speaking, factors that influence medical students’ career pursuits range from personality and personal attributes [3-5], to gender differences [6], to issues of prestige and income [7-11]. The practice of EM involves rapid decision-making in an acute setting, a broad knowledge base, and an ability to multi-task [12]. Lifestyle, varied scope of practice, emphasis on acute care and previous experiences in emergency care influence students’ interest in EM [3-4,13].

No studies were identified that followed students throughout their schooling to assess how their attitudes towards EM change over time. Further, research in this area does not tend to be exploratory or qualitative. We aimed to describe the changing proportion of students who consider EM over the course of undergraduate training, explore factors that influence students’ decisions and contextualize these factors by seeking perspective from career emergency physicians.

## Materials and methods

Setting

This study used a longitudinal survey to examine trends in students’ self-identified interest in EM as a career over the course of four years of undergraduate medical training at Memorial University of Newfoundland. Between 1999 and 2008, the Career Choices survey was distributed to medical students in the graduating classes of 2000-2008.

The Career Choices study was reviewed and approved by the institutional ethics review board. Descriptive findings about students’ interest in family medicine as a career have previously been published [14].

Design

A paper-based survey was designed to ask about all specialties for which graduating students are eligible through CaRMS, including EM. For each specialty, students could indicate “yes, interested,” “no, not interested,” or “don’t know.” They were not asked to rank their choices. Students provided their rationale for and against the specialty in an open-ended format.

Data collection

The survey was administered at five iterations over four years of training to capture students’ career interests. The five data-collection points (DCPs) were as follows: 1) within two months of starting medical school in first year; 2) at the end of first year; 3) at the end of second year; 4) at the start of clerkship; and 5) near the end of fourth year (after completing the residency match). The first year class was surveyed twice to obtain their initial impressions at the beginning of the year, prior to any medical training and their career choices at the end of their first year after exposure to the curriculum. Thereafter, each class was surveyed once. The classes of 2003-2006 completed all five iterations while the other classes had fewer DCPs.

Students’ responses were anonymous but coded with identification numbers to enable tracking of students’ changing career interests over time. Students usually completed and handed in the surveys during a class gathering. When unanticipated timetable changes resulted in missing the opportunity to collect surveys face-to-face, surveys were mailed with return-address envelopes. Return rates from mailings were poor.

Analysis

Quantitative data were tabulated across graduation years and DCP. Comparisons between specialty choice and gender were tested using Fisher’s test and the effect size was calculated using odds ratios (ORs). The effect of endurance (how likely a student selecting a specialty in first year was to select it in fourth year) was calculated using logistic regressions and compared using a Forest plot.

The qualitative analysis was guided by grounded theory to systematically generate conceptual categories or themes from the data from “the ground up” [15]. Two authors independently reviewed the qualitative data at least three times and coded them based on recurring themes that arose relating to EM as a career choice. The data review involved gaining a general understanding of the survey responses, followed by “open coding” (identifying, naming and categorizing the data into specific themes), and lastly “axial coding,” (relating categories to one another to fit the data into more connected themes). All themes related to the core category of EM as a career choice (i.e., “selective coding”). Independently, two authors systematically coded by hand in conjunction with spreadsheets to manage categories and track sentinel quotes to exemplify each theme. Subsequently, these two authors met to ensure consistency, compare relationships amongst the themes and explore incongruous ideas.

Lastly, we conducted interviews with academic emergency physicians at Dalhousie University between October 2015 and January 2016. This was an “audience review as credibility triangulation,” i.e., an audience who may use the study findings (emergency physicians) had the chance to review findings and add perspective [15]. Consenting physicians were provided with a brief summary of the themes identified in our analysis. Using a semi-structured interview guide, emergency physicians had an opportunity to clarify, agree, or disagree with themes based on their perspectives and experiences of a career in EM and consider how students’ career perceptions might influence recruitment and subsequent residency training. In addition to notes, the encounters were audio recorded, transcribed and de-identified. Again, we systematically coded by hand in conjunction with spreadsheets to manage categories and track sentinel quotes to exemplify each theme and subsequently met to ensure consistency, compare relationships amongst the themes and explore incongruous ideas. The "audience review as credibility triangulation" aspect of the study was separately reviewed and approved by our institutional research ethics board.

## Results

Medical student career choices survey

There were 1480 surveys completed by 543 students in the graduating classes of 2000-2008; 1281 surveys had written responses and 759 written survey comments pertained to EM. Table 1 presents the overall choices of medical students for EM as well as five other specialties for comparison (Anesthesia, General Surgery, Internal Medicine, Pediatrics and Family Medicine), stratified by gender. There was a significant effect of gender, with males more likely to select EM (OR = 1.5, 95% CI: [1.2, 2.0]).

**Table 1:** Specialty selections across years, overall and by gender. The p-value is calculated between genders across the categories using a Fisher’s test. The odds ratio (OR) indicates the odds of selecting Yes vs. selecting No (i.e., of ignoring the Don’t Know choices) for a male vs. female. CI = Confidence Interval

Specialty	Gender	"No" n (%)	"Don't know" n (%)	"Yes" n (%)	p-value	OR [95% CI]
Emergency Medicine	Male	257 (43.7)	144 (24.5)	187 (31.8)	<0.01	1.50 [1.16, 1.96]
	Female	403 (50.4)	201 (25.2)	195 (24.4)		
Anesthesia	Male	351 (59.7)	164 (27.9)	73 (12.4)	<0.01	1.82 [1.25, 2.65]
	Female	559 (70)	176 (22)	64 (8)		
Family Medicine	Male	252 (42.9)	104 (17.7)	232 (39.5)	<0.01	0.52 [0.41, 0.66]
	Female	249 (31.2)	107 (13.4)	443 (55.4)		
Internal Medicine	Male	244 (41.5)	152 (25.9)	192 (32.7)	0.02	1.44 [1.11, 1.87]
	Female	382 (47.8)	208 (26)	209 (26.2)		
Pediatrics	Male	322 (54.8)	117 (19.9)	149 (25.3)	<0.01	0.58 [0.45, 0.75]
	Female	373 (46.7)	129 (16.1)	297 (37.2)		
General Surgery	Male	288 (49)	137 (23.3)	163 (27.7)	<0.01	2.99 [2.24, 4.02]
	Female	556 (69.6)	138 (17.3)	105 (13.1)		

**Figure 1:**
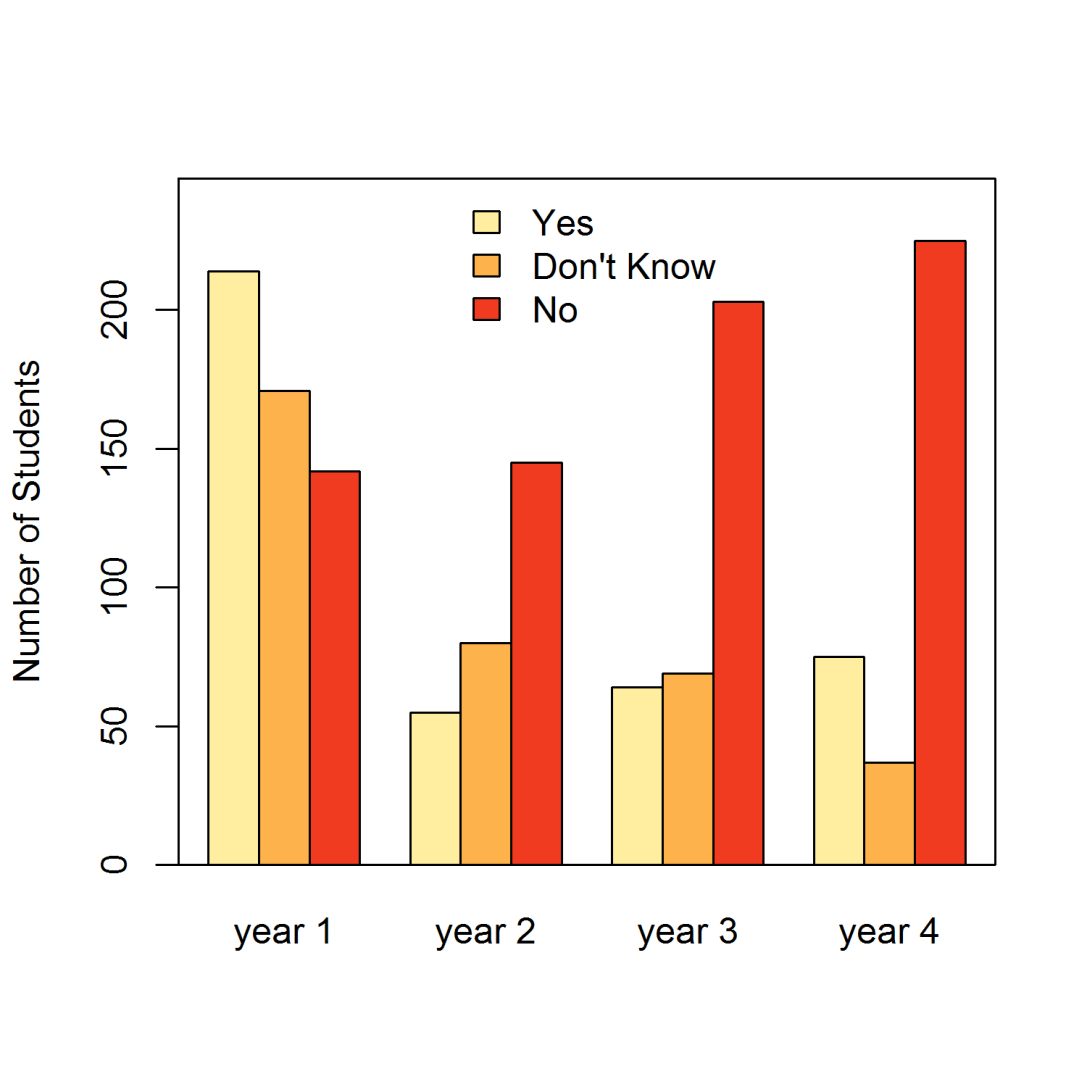
Interest in Emergency Medicine as a career by year.

**Figure 2:**
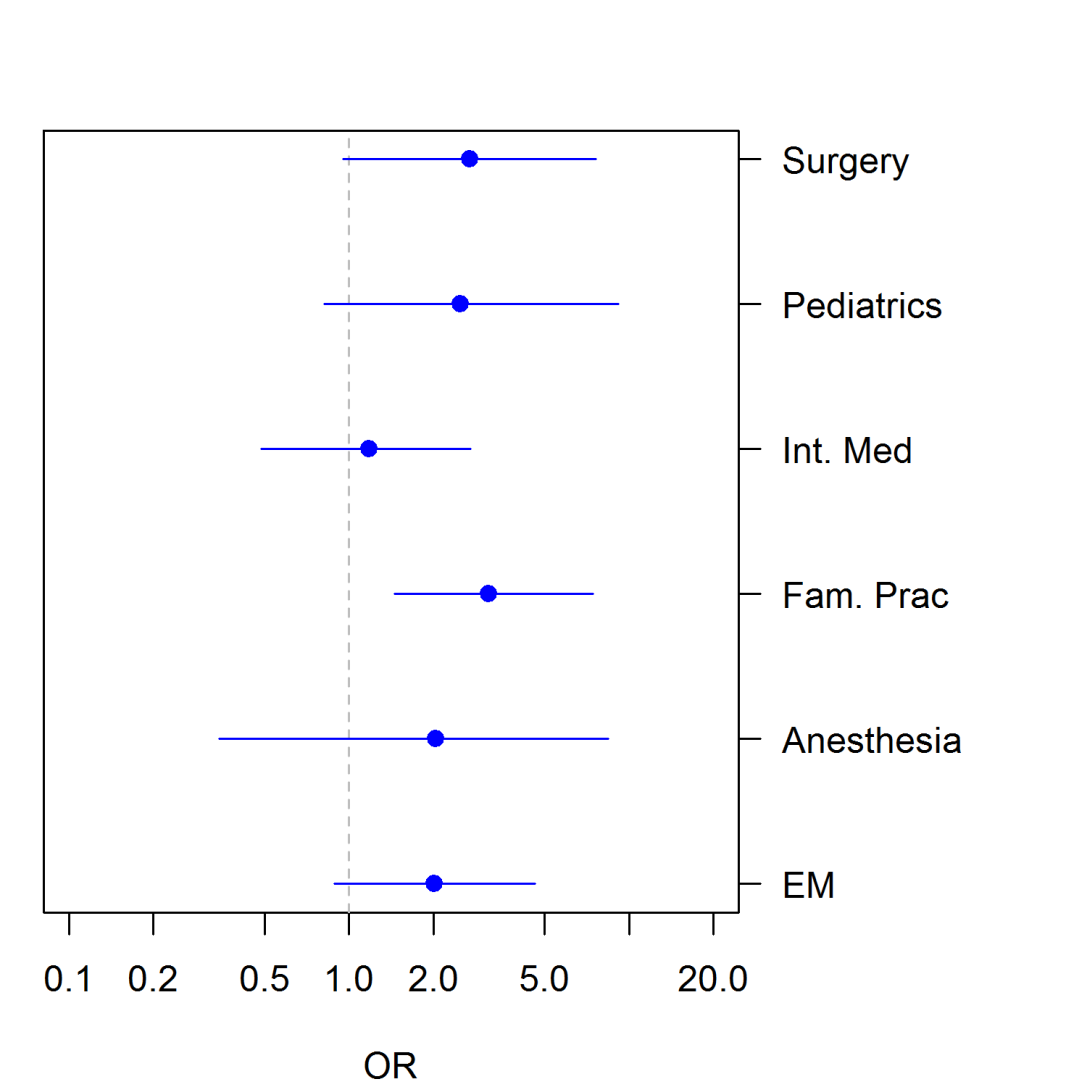
Forest plot of odds ratios (OR) with 95% confidence intervals (CI) for endurance of career choice for six specialties. Int. Med = Internal Medicine; Fam. Prac = Family Medicine; EM = Emergency Medicine

Figure 1 presents medical students’ selection of EM over their curriculum. To calculate endurance, we classified students according to whether their specialty choice in first year matched their choice in fourth year. EM’s endurance (OR = 2.0, 95% CI: [0.9, 4.6]) was lower than family medicine (OR = 3.1, 95% CI: [1.4, 7.4]) but higher than internal medicine (1.2, 95% CI: [0.5, 2.7]). Only family medicine demonstrated a statistically significant endurance metric (Figure 2).

We identified six major and four minor themes describing factors that influence medical students’ interests in an EM career (Tables 2, 3). The major themes (Table 2) included: lifestyle, nature of the work, perception of the work, previous experiences, passion/personal philosophy, and the option of training in EM as a family physician (College of Family Physicians of Canada Emergency Medicine program: CCFP-EM). The minor themes included: media, mentorship/modelling, residency curriculum and admissions, and outside influences/opinions. Illustrative quotes are listed in Table 3. Overall, the biggest drawbacks for EM were cited as lack of follow-up care and lack of exposure; the biggest pull factors were clinical variety, congruence with pre-med experiences, and positive clinical experiences.

**Table 2:** Identified major themes and representative quotes from the Career Choices survey relating to Emergency Medicine (EM) as a career. DCP = Data Collection Point

Major themes	Representative participant quotes
Lifestyle: Balance; Hours; Stress	“Not conducive to family life (want children); long hours, high stress, not a set schedule.” (Class of 2001, DCP4) “Great lifestyle, very interesting.” (Class of 2000, DCP5)
Nature of the work: Variety; Diverse patient population; Shift work; Trauma situations; Lack of continuity of care	“Ability to encounter a variety of situations, constantly changing case load.” (Class of 2005, DCP1) “Shift work that is intense = burn out after long term.” (Class of 2005, DCP2) “…No long term care (except the returning drunks), no continuity of care.” (Class of 2000, DCP5)
Perception of work: Challenging; Interesting/exciting	“Seems like long hard hours!” (Class of 2005, DCP1) “I feel it would be exciting and challenging and would not get boring…” (Class of 2003, DCP1) “Terror, punctuated by boredom - much preferable [than the opposite].” (Class of 2003, DCP2) “Blood and guts are cool and it sounds challenging with a broad spectrum of skills required.” (Class of 2005, DCP2)
Previous experiences: Pre-med; Electives/rotations	“I volunteer with St. John Ambulance, and have experienced pre hospital emergency care – so I find it quite interesting.” (Class of 2003, DCP1) “I had a good experience on the rural visit in the ER. I didn't think I would like it as much as I did.” (Class of 2005, DCP2)
Passion/personal philosophy	“I thrive in the most stressful situations and feel that emergency medicine may be where I am best suited and will be happiest.” (Class of 2004, DCP1) “Don't think my personality is suited to life or death decisions that need to be made in milliseconds on a daily basis. Extremely high stress.” (Class of 2005, DCP3)
College of Family Physicians Canada (CFPC) Emergency Medicine Program	“Would like to do family [medicine] and a year of emergency because very practical for rural practice or international practice.” (Class of 2003, DCP5) “Plan to complete the CCFP (EM) program. Enjoy the variety + flexibility of tailoring your practice to your needs.” (Class of 2000, DCP5)

**Table 3:** Identified minor themes and representative quotes from the Career Choices survey relating to Emergency Medicine (EM) as a career. DCP = Data Collection Point

Minor themes	Representative participant quotes
Media	“Immediate gratification. I saw it on TV.” (Class of 2006, DCP3) “The fast paced work environment…especially as depicted by ER.” (Class of 2007, DCP1)
Mentorship/modeling	“I did an elective, both Dr. X and Dr. Y were excellent teachers.” (Class of 2001, DCP5)
Residency curriculum & admissions	“Specialty too competitive to get into. Students are starting in first year to try to get into this specialty.” (Class of 2003, DCP5) “Specialty training is too specialized and to keep up skills need to work in large center…” (Class of 2000, DCP5)
Outside influences & opinions	“Aunt is an emergency room doctor, doesn't have a lot of time for family, etc. Not appealing.” (Class of 2008, DCP1) “I have known wonderful people who are emergency physicians although they wouldn't recommend it. I love the spontaneous interaction with people.”(Class of 2004, DCP1)

Lifestyle was consistently cited throughout the DCPs; it was described by students in both a general sense (“great lifestyle” or “bad lifestyle”) or more specifically, relating to hours and stress. Interestingly, there was no consensus as to whether the lifestyle of EM was more beneficial (pull factor) or negative (push factor). Statements made about the lifestyle of EM prior to any curricular exposure to the specialty were often negative. For example: “Haven't had any exposure yet, want a family, so lifestyle may be an issue” (Class of 2002, DCP3). In most cases when EM lifestyle was described as a pull factor, it was also coupled with other pull factors, such as practice variety and diverse patient populations.

Nature of the work was cited as a prominent theme including objective elements of the job such as variety, diverse patient population, shift work, trauma, and no/minimal follow-up care. Variety of cases was cited as an independent pull factor (“Enjoy the variety of [patient] cases…”) but was more often coupled with other pull factors (“Exciting, variety of medicine seen, fast pace”). In contrast, lack of follow-up care was frequently cited as a push factor and appeared to be more influential during clerkship.

Perception of the work was also a major theme, referring to how students subjectively described EM. Common descriptors across DCPs were: interesting, exciting, challenging, and fast-paced. With the exception of the term “exciting,” it could not be clearly identified as a push or pull factor. Perceptions earlier on (i.e., DCP1 and DCP2) evolved during training to incorporate more objective comments about the nature of the work, or ideas about personal philosophy. One participant highlights this transition:

“High paced, exciting” (DCP1); “Exciting--yet stressful” (DCP2); “Variety of work; shift work is attractive” (DCP3); “Exciting. Variety of problems” (DCP4); “Wide range of clinical problems” (DCP5).

Previous experiences with EM focused on two timeframes: pre-medical school life experiences and curricular experiences (electives and rotations). Pre-med experiences were largely positive influences towards a career in EM. As students progressed through their schooling, pre-med life experiences appeared to become less influential. Students mentioned the influence of their curricular experiences (e.g., electives or rural visits) as early as DCP2 (end of first year). These early curricular experiences were rated as largely positive. In contrast, some participants had minimal exposure to EM in their training and cited this as a rationale for their uncertainty about the specialty.

Many students indicated interest in the CCFP-EM program. Students felt that the CCFP-EM program would provide more opportunities, increased flexibility, or broader geographic applicability (rural or international). Interest in the CCFP-EM program was more prominent at later DCP4 and DCP5.

Emergency physician interviews

**Figure 3:**
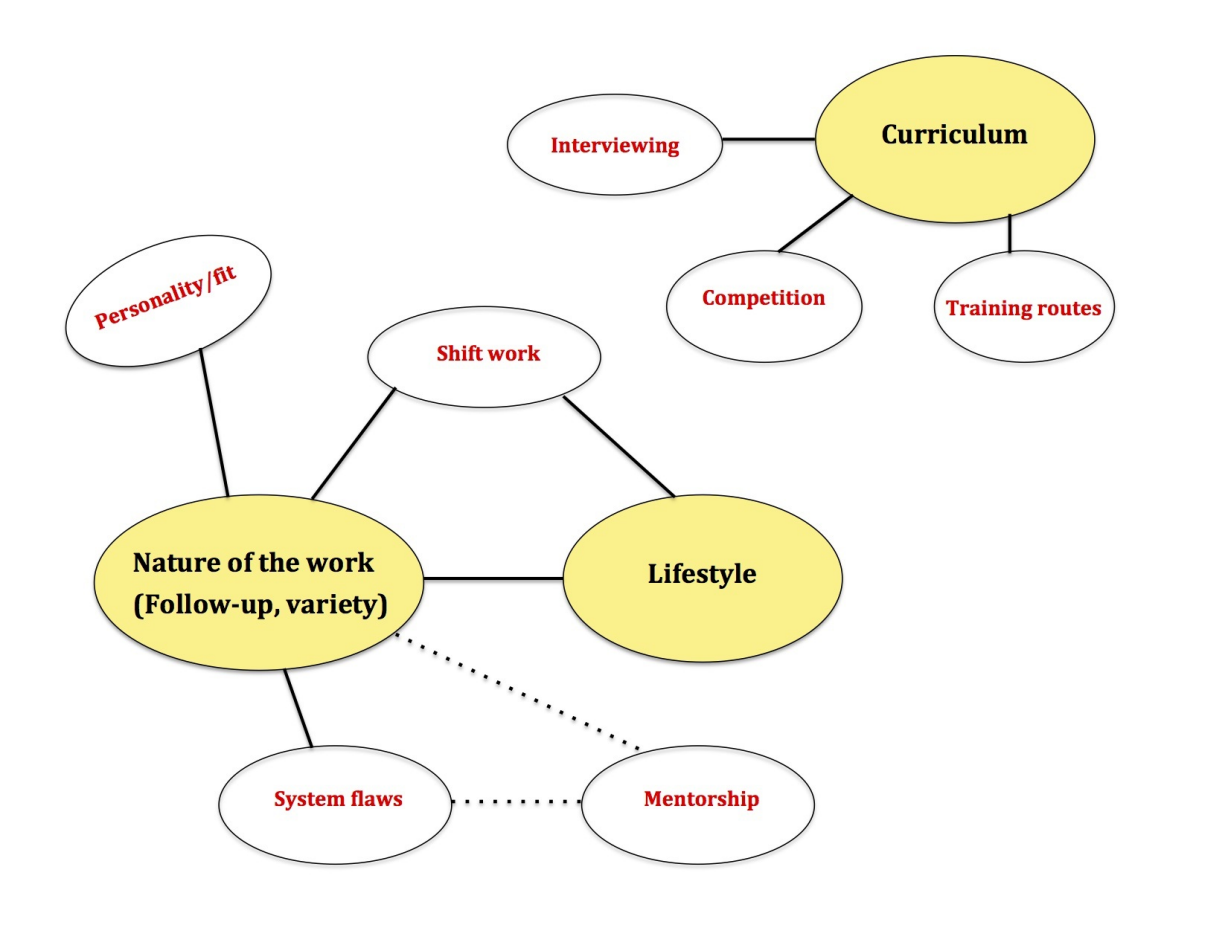
Identified themes from Emergency Medicine (EM) physician interviews. Note: lines represent connection between themes; yellow represents the three major themes.

We interviewed five academic emergency physicians: three interviews were face-to-face and two were performed over the telephone. Analysis revealed interconnected themes that fell within one of three larger categories: nature of the work, lifestyle, or curriculum (Figure 3).

Each interviewee discussed students’ misconceptions about EM, particularly about the lifestyle of EM and nature of the work. These emergency physicians consistently described the lifestyle of EM as better than most specialties, because of flexible scheduling and the ability to achieve positive work-life balance:

“I would argue that these [EM physicians] really have a great lifestyle, work life balance. I think we need to be highlighting these things with our learners and saying, yes you can work 80 hours a week and some of that is choice, how you set up your practice… and here are some of the things you may consider doing to exert some control while still being a team player.” (Participant 4a)

Commentary on shift work related equally to lifestyle and its role in the nature of the work/emergency department. Physicians commented about the duality of shift work as both a “blessing and burden,” allowing scheduling flexibility but also attributing it, in part, to burnout:

“Because of the shift load there’s a prediction of burn out… but it’s not because of the long hours.” (Participant 2a)

“...what do I like best about the specialty? That was the shift work and like least is the shift work.” (Participant 4a)

Emergency physicians also brought up the concept of “system flaws:” aspects of EM that students are likely not privy to when considering a career. This particularly related to the nature of the emergency department, as well as the effect of shift work:

“The perception from the students is that the emergency physician is always doing resuscitative thoracotomies and central lines and intubating people, whereas really those are very uncommon...most of the time you’re looking at people with vague, unexplained abdominal pain or weakness or shortness of breath.” (Participant 2a)

Physicians related much of this discussion to the role of mentorship in providing an accurate depiction of a career in EM. They also commented on the interplay between system flaws and off-service rotations, and how this may frame a career in EM:

“We practice in a glass house…So we’re always being scrutinized.” (Participant 1a)

Overall, while there was disagreement between physicians and students as to the perception of the work and the lifestyle in EM, they agreed with the statements on variability and lack of follow-up care.

In discussion about resident recruitment to EM, the consensus was to use these themes during the interview process, and ensure students are supported in their choices once they become residents:

“How do we make sure that once people are in the specialty that we keep giving them what they need. So how do we make sure that...the training programs and beyond are preparing them for the right types of careers and sustaining them in those careers.” (Participant 3a)

With regards to candidate selection, physicians discussed the role of competition and its effect on recruitment. They expressed concern that with increasing competition, students are asked to make decisions earlier in their schooling, and are thus not as well-rounded by the time they reach resident training programs. In general, they did not see the need to use these themes to aid recruitment since interest in EM is high; it is not about recruiting more students, but rather, ensuring that the students who select a career in EM are doing so for the right reasons, i.e., their rationale aligns with the true nature of emergency medicine:

“It would be very enlightening to include similar questions in the interview process to know if [applicants] are getting into it for the right reason. If they’re entering into a specialty and they have really no clear ideas as to what it’s like, they may not end up being very happy in it.” (Participant 1b)

## Discussion

This study provides a unique perspective as to what influences medical students’ career choices with respect to EM through an exploratory and prospective design that followed students throughout their schooling to assess how their attitudes towards EM changed over time. While most past studies have investigated career choice in general [16-17], few have focused on emergency medicine [4,13,18]. Scott, et al. examined Canadian medical student interest in EM at the start of their schooling, and personal characteristics associated with primary interest in EM [4]. Our study is longitudinal and follows students throughout their schooling, largely assessing influential factors rather than personal characteristics.

Women tend to be underrepresented in EM careers [19]. Although women in our study were significantly less likely to express interest in an EM career, the reasons are not clear. Negative gender stereotypes may contribute to this trend [20].

EM has been described as a ‘controllable lifestyle’ specialty, in which one has more control over their work hours [21]. Controllable lifestyle has been highlighted as a key pull factor in choosing a specialty [13,22]. A Japanese study found that students who sought “work-life balance” were less likely to pursue an EM career [23]. In our study, students commonly referenced lifestyle but didn’t clearly indicate whether the EM lifestyle was positive or negative. Clinical variety, on the other hand, was clearly a motivating factor. A large Finnish study of the rationale behind choosing to enter medical school found that a wide range of professional activities were important motives for entering students [24]. It appears that clinical range and variety are also influential in choosing an EM career.

Students’ views of EM tended to shift throughout their schooling, from more subjective to more objective concepts as they gained curricular experience. An American study of the impact of a mandatory EM rotation during clerkship found no significant changes in students’ overall perceptions of EM [25]. In contrast, a British study found that clinical attachments significantly correlated with interest in an EM career [26]. Our study found that clerkship experiences were largely positive which may relate more to students’ pre-existing interests. With growing competition for EM residency in Canada, students often feel pressured to declare their interests early on through choosing non-mandatory experiences, perhaps contributing to the “endurance” of EM as a choice or a tunnel vision effect.

The EM physician interviews highlighted the impact of “system flaws.” These flaws emerge through the hidden curriculum where students learn lessons that are not openly intended [27]. Many students rotating in EM are keen to see trauma, resuscitations and the rarities of EM, but may not initially show as much interest in more common clinical presentations. As such, medical students may be steered toward “exciting" cases, potentially resulting in highly positive experiences and skewed perceptions as to the degree to which “exciting” cases contribute to an EM career. With concerns about emergency physician attrition and burnout, an accurate perception of EM is important [2,28-29]. A recent meta-analysis found that mentoring positively affects career planning and satisfaction [30]. Mentoring and more holistic EM rotations may better prepare students for the realities of an EM career.

Future directions

Themes from the Career Choices survey could be useful at both the undergraduate curricular and residency interview level. Offering early exposure to EM may help students contemplate and assess specialty characteristics, career trajectory and “fit.” Ongoing career services might be beneficial in reducing tunnel vision and encouraging students to self-reflect.

As mentioned during the physician interviews, the themes could be useful during candidate selection to assess students’ rationale for an EM career i.e. determining whether students understand the nature of the career to which they are applying. With the prevalence of skewed perceptions amongst undergraduates, and the increasing competitiveness of EM, demonstrated understanding of the scope and lifestyle of EM could be a predictor of success in residency training.

Limitations

The student data in this study was collected at a single site from a school that did not have a residency training program in EM. These students may have different experiences compared to medical schools with established EM programs. The Career Choices survey collected students’ perceptions in an open-ended format where students largely wrote short comments. The nuances of how these factors impacted their decisions could not be explored in this format. Interviewing EM faculty from a different institution for “credibility triangulation” helped to identify any incongruences.

## Conclusions

Males were significantly more likely to express interest in an EM career. Medical students’ opinions towards EM tended to shift throughout their schooling, particularly, the perception of the work. Medical students’ perceptions differ from that of experienced emergency physicians. Medical schools may be able to improve clinical exposure and provide more informed counselling or mentoring with respect to emergency medicine.
